# Supplementary material for: A Systematic Review of Literature on the Association Among Sleep, Cortisol Level and Cardiovascular Health Within the Healthcare Shift Worker Population
Source: Biomedicines. 2025 Oct 17;13(10):2539. doi: 10.3390/biomedicines13102539 (PMC12561395; doi:10.3390/biomedicines13102539)
Supplement: Supplementary file 1 [file biomedicines-13-02539-s001.zip › biomedicines-3894294-supplementary.pdf]

Table S1: Quality assessment of the studies included in the systematic review.

No	Author, Year	Question assessing—JBI cross sectional studies								Yes (%)
		1	2	3	4	5	6	7	8	
1	Vivarelli et al., 2023 [24]	Y	Y	Y	Y	U	U	Y	Y	87.5
2	Lowson et al., 2013 [30]	U	Y	Y	Y	U	U	Y	Y	81.25
3	Bani-Issa et al., 2020 [27]	Y	Y	Y	Y	Y	Y	Y	Y	100
4	Tsai et al., 2019 [28]	Y	Y	Y	Y	Y	Y	Y	Y	100
5	Chang et al., 2018 [29]	Y	Y	Y	Y	Y	Y	Y	Y	100
6	Roy et al., 2023 [35]	Y	Y	Y	Y	U	U	Y	Y	87.5
7	Ljevak et al., 2020 [26]	Y	Y	Y	Y	U	U	Y	Y	87.5
8	Zhang et al., 2023 [23]	Y	Y	Y	Y	Y	Y	Y	Y	100
9	Minelli et al., 2021 [25]	Y	Y	Y	Y	Y	Y	Y	Y	100
10	Panwar et al., 2024 [33]	Y	Y	Y	Y	U	U	Y	Y	87.5
11	Hsu et al., 2021 [16]	Y	Y	Y	Y	Y	Y	Y	Y	100
12	Silva-Costa et al., 2015 [32]	Y	Y	Y	Y	Y	Y	U	Y	93.75
13	Lajoie et al., 2015 [31]	Y	Y	Y	Y	Y	Y	Y	Y	100

Y= Yes; N=No; U=Unclear

No	Author, Year	Question assessing—RoB 2 tool for randomized control trial					Overall
		1	2	3	4	5	
1.	Liao et al., 2025 [34]	Low	Low	Low	Low	Low	Low risk of bias

JBI Cross sectional studies assessment

Questions:

1. Were the criteria for inclusion in the sample clearly defined?
2. Were the study subjects and the setting described in detail?
3. Was the exposure measured in a valid and reliable way?
4. Were objective, standard criteria used for measurement of the condition?
5. Were confounding factors identified?
6. Were strategies to deal with confounding factors stated?
7. Were the outcomes measured in a valid and reliable way?
8. Was appropriate statistical analysis used?

RoB 2 assessment for randomized control trial

Questions

1. Domain 1: Risk of bias arising from the randomization process;
2. Domain 2: Risk of bias due to deviations from the intended interventions (effect of assignment to intervention);
3. Domain 3: Risk of bias due to missing outcome data;
4. Domain 4: Risk of bias in measurement of the outcome;
5. Domain 5: Risk of bias in selection of the reported result.
